# The undiscovered biosynthetic potential of the Greenland Ice Sheet microbiome

**DOI:** 10.3389/fmicb.2023.1285791

**Published:** 2023-12-12

**Authors:** Ate H. Jaarsma, Athanasios Zervas, Katie Sipes, Francisco Campuzano Jiménez, Andrea Claire Smith, Liessel Victoria Svendsen, Mariane S. Thøgersen, Peter Stougaard, Liane G. Benning, Martyn Tranter, Alexandre M. Anesio

**Affiliations:** ^1^Department of Environmental Science, Aarhus University, Roskilde, Denmark; ^2^German Research Centre for Geosciences, Helmholtz Centre Potsdam, Potsdam, Germany; ^3^Department of Earth Sciences, Freie Universität Berlin, Berlin, Germany

**Keywords:** supraglacial habitats, extremophiles, metagenomic sequencing, biosynthetic gene clusters, genome mining, bioprospecting

## Abstract

The Greenland Ice Sheet is a biome which is mainly microbially driven. Several different niches can be found within the glacial biome for those microbes able to withstand the harsh conditions, e.g., low temperatures, low nutrient conditions, high UV radiation in summer, and contrasting long and dark winters. Eukaryotic algae can form blooms during the summer on the ice surface, interacting with communities of bacteria, fungi, and viruses. Cryoconite holes and snow are also habitats with their own microbial community. Nevertheless, the microbiome of supraglacial habitats remains poorly studied, leading to a lack of representative genomes from these environments. Under-investigated extremophiles, like those living on the Greenland Ice Sheet, may provide an untapped reservoir of chemical diversity that is yet to be discovered. In this study, an inventory of the biosynthetic potential of these organisms is made, through cataloging the presence of biosynthetic gene clusters in their genomes. There were 133 high-quality metagenome-assembled genomes (MAGs) and 28 whole genomes of bacteria obtained from samples of the ice sheet surface, cryoconite, biofilm, and snow using culturing-dependent and -independent approaches. AntiSMASH and BiG-SCAPE were used to mine these genomes and subsequently analyze the resulting predicted gene clusters. Extensive sets of predicted Biosynthetic Gene Clusters (BGCs) were collected from the genome collection, with limited overlap between isolates and MAGs. Additionally, little overlap was found in the biosynthetic potential among different environments, suggesting specialization of organisms in specific habitats. The median number of BGCs per genome was significantly higher for the isolates compared to the MAGs. The most talented producers were found among Proteobacteria. We found evidence for the capacity of these microbes to produce antimicrobials, carotenoid pigments, siderophores, and osmoprotectants, indicating potential survival mechanisms to cope with extreme conditions. The majority of identified BGCs, including those in the most prevalent gene cluster families, have unknown functions, presenting a substantial potential for bioprospecting. This study underscores the diverse biosynthetic potential in Greenland Ice Sheet genomes, revealing insights into survival strategies and highlighting the need for further exploration and characterization of these untapped resources.

## 1 Introduction

Bacteria are capable of producing a myriad of chemical structures that are beneficial to their own survival (Gavriilidou et al., 2022). Some of these natural products have antimicrobial activity and are employed to wage chemical warfare with competing microorganisms (Ghoul and Mitri, 2016). Therefore, as the world searches for novel antimicrobial therapeutics in light of the antimicrobial resistance crisis (Murray et al., 2022), there is a large interest in the chemical diversity of microbially produced natural products. In addition to antibiotics, the chemical diversity of microbial natural products has inspired numerous anticancer drugs (Cragg and Newman, 2013) and antivirals (Ma et al., 2020), further illustrating the value of drugs inspired by natural biosynthesis. In addition to therapeutics, natural products can inspire the synthesis of many other bioactive compounds (Cordier et al., 2008).

Under-explored extremophiles from cryospheric environments may harbor an untapped reservoir of chemical diversity due to their unique adaptations to extreme conditions, such as adaptations to frequent freeze-thawing conditions, high UV radiation and low nutrient levels. Glaciers and ice sheets can harbor a diverse range of microorganisms. The bacterial community of the cryosphere is dominated by Proteobacteria and Bacteroidetes (Bourquin et al., 2022). The average microbial abundance in surface meltwaters is regionally consistent, about 10^4^ cells mL^−1^ (Stevens et al., 2022). Glaciers and ice sheets are thus considered biomes, which are mainly microbially driven (Anesio et al., 2017). Over 200 natural products have been discovered already from polar organisms (Tian et al., 2017). Furthermore, predicted novel chemical diversity has, for example, been found in actinomycetes from polar marine soil (Soldatou et al., 2021), isolates from the Canadian Arctic (Marcolefas et al., 2019), and genomes from Tibetan glaciers (Liu et al., 2022).

In recent years, the Next Generation Sequencing (NGS) revolution (Reuter et al., 2015) has made it cheaper and easier to gain access to biosynthetic potential through genomic information. Third-generation sequencing technologies (PacBio, Oxford Nanopore Technologies) have subsequently revolutionized the field, and are now crucial for deciphering positions, orientations, etc., during the sequencing of complete genomes (Athanasopoulou et al., 2022). Metagenome sequencing can be used to obtain metagenome-assembled genomes (MAGs) without the need to have the organism in culture. Furthermore, a range of bioinformatics genome mining tools are available to detect and analyze gene clusters responsible for the production of natural products (Tracanna et al., 2017; Medema et al., 2021). The “antibiotics and secondary metabolite analysis shell” (antiSMASH) is the most used tool for detecting biosynthetic gene clusters (BGCs) within microbial genomes (Blin et al., 2021). Using manually curated rules, antiSMASH is able to identify a range of different biosynthetic pathways and provides the regions they are encoded in as output. Tools like the “biosynthetic gene similarity clustering and prospecting engine” (BiG-SCAPE) (Navarro-Muñoz et al., 2020) facilitate subsequent exploration of large datasets, for instance from mining a large number of genomes, by enabling the construction of sequence similarity networks of the resulting BGCs from thousands of genomes at once. The MIBiG (Minimum Information about a Biosynthetic Gene cluster) repository can subsequently be used for comparison of these BGCs to ones previously characterized experimentally (Terlouw et al., 2023).

Despite these advancements, it is speculated that a substantial portion of the chemical diversity present in nature remains undiscovered (Scott and Piel, 2019). Many organisms, representing diverse branches on the tree of life, have not yet been successfully cultured in the laboratory (Hug et al., 2016). Although over 50,000 MAGs from various regions around the world have been cataloged (Nayfach et al., 2020), genomes of microbes from polar environments are still under-represented.

The Greenland Ice Sheet is a microbially driven cryospheric habitat containing potentially under-explored biodiversity. In summer, the melting ice surface contains a proliferation of eukaryotic glacier ice algae, which causes the ice surface to darken by virtue of their pigmentation (Cook et al., 2020). The pigmented glacier ice algae, *Ancylonema alaskanum* and *Ancylonema nordenskiöldii*, are the main species driving this biological albedo reduction (Lutz et al., 2018). However, alongside the glacier ice algae, a diverse community of bacteria, fungi, and viruses is associated with the algal blooms. Proteobacteria, Actinobacteria, and Bacteroidetes are typically the dominant bacterial phyla (Jaarsma et al., 2023), and Chytridiomycota fungi potentially play an important role in infecting and decomposing of glacier ice algae (Perini et al., 2022). Furthermore, cryoconite holes and snow form distinct habitats housing unique microbial communities (Anesio et al., 2017).

This study aims to investigate the biosynthetic potential present in bacterial genomes derived from supraglacial habitats on the Greenland Ice Sheet. Doing so, we seek to shed light on the potential to produce antimicrobials, as well as other natural products, that may aid their survival in the cryosphere. A genome collection was previously obtained using culturing-dependent and -independent methods, including 133 high-quality MAGs and 28 isolate draft genomes (Jaarsma et al., 2023). Here, we describe the production of complete closed genomes from those isolates, in order to mine them together with our collection of MAGs. The distribution of biosynthetic gene clusters (BGCs) was analyzed across different sample types (surface ice, cryoconite, biofilm, snow), and a comparison was made between isolates and MAGs. Additionally, the presence of BGCs in various phyla was examined to identify organisms with high potential for natural product production.

## 2 Materials and methods

### 2.1 Sample collection

**Figure 1:**
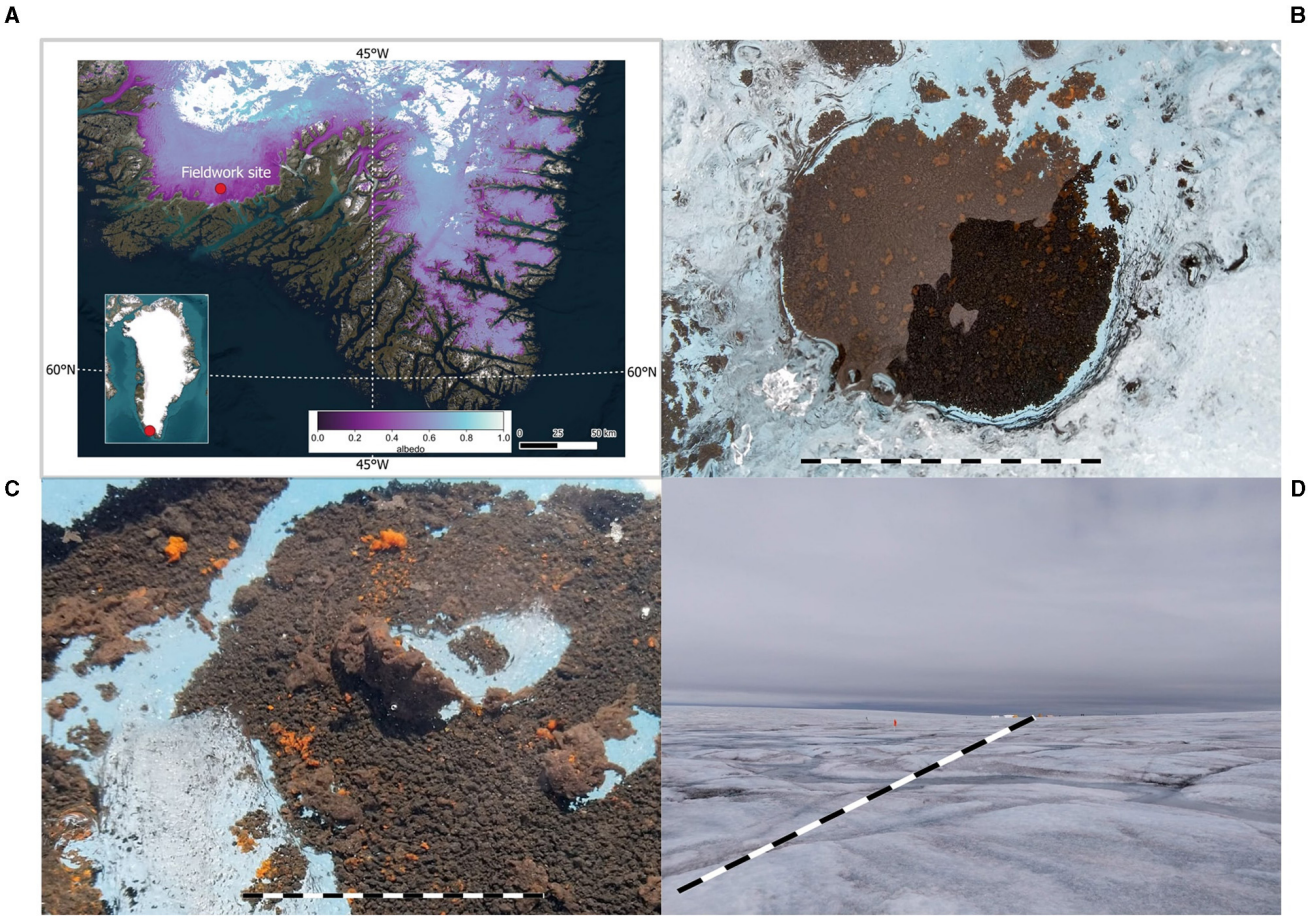
**(A)** Fieldwork site on the southwest margin of the Greenland Ice Sheet. Map layers were created using ©Esri, Maxar, Earthstar Geographics, and the GIS User Community. EPSG:3411. The map shows the average albedo between 13-7-2021 and 25-8-2021 generated using a 30 m harmonized satellite albedo (Feng et al., 2023). **(B–D)** examples of supraglacial habitats sampled in this study; cryoconite [**(B)**, scale bar = 20 cm], biofilm in cryoconite hole [**(C)**, scale bar = 15 cm], dark ice surface [**(D)**, scale bar = approx. 150 m].

Sample collection was carried out during the July–August 2021 Deep Purple expedition (https://www.deeppurple-ercsyg.eu/). The camp was established in the south of the Greenland Ice Sheet, situated approximately 7.5 km from the margin at coordinates 61.10138895, -46.8481389, and at an elevation of 617 m a.s.l. (Figure 1A).

Environmental samples from both the dark ice surface and cryoconite holes were gathered over a 100 × 100 m area (Figures 1B–D). Ice samples were obtained by scraping roughly two vertical cm of the dark ice surface from 30 patches within the sample area using an ice axe. These samples were stored in sterile 4L Whirl-pak bags and allowed to melt at an ambient temperature of 5-10 °C. Cryoconite sediment was collected from 30 different cryoconite holes within the designated area using a polycarbonate aquarium pipette. A total of 18 kg of surface ice and 3.5 kg of cryoconite sediment were collected. The ice and cryoconite samples were combined and homogenized within their respective sterile bags. Subsamples of both ice and cryoconite samples were taken in 50 mL tubes and kept cool during transportation to the lab.

Three 4 mL cryotubes were filled with cryoconite sediment for DNA extraction. In the case of ice samples, biomass for DNA extraction was obtained by filtering three technical replicates of 500 mL of melted ice through Sartorius cellulose nitrate filters (0.2 μm). These filters were stored in 4 mL cryotubes after being rolled up. Additional samples, including a viscous suspended biofilm from a cryoconite hole and a red snow sample, were also collected. These samples were gathered in 50 mL tubes and kept cool during transportation back to the lab. A subsample of the biofilm was taken for DNA extraction. All samples designated for DNA extraction were frozen in the field camp and maintained at -20 °C during transport to Aarhus University, Roskilde, Denmark.

Bacteria were isolated in axenic cultures from cryoconite, ice, biofilm, and snow samples using Petri dishes and *in situ* culturing setups [described in detail in Jaarsma et al. (2023)]. Cultures were maintained on Reasoner's 2 agar (R2A) (Linde et al., 2000) (Alpha Biosciences) at 5 °C.

### 2.2 Metagenome sequencing

DNA extraction for shotgun metagenome sequencing was carried out on one biofilm sample and three technical replicates of cryoconite sediment and filters containing ice biomass using a DNeasy PowerLyzer Power Soil kit (Qiagen). The seven libraries were generated using the Ultra FS II DNA Library Prep Kit for Illumina (New England Biolabs, Ipswich, USA) in accordance with the manufacturer's protocol. These libraries were equimolarly combined and examined for insert size distribution and the presence of adapter-primer dimers using a TapeStation 4150 with a D1000 DNA ScreenTape (Agilent Technologies). The library was diluted and denaturated following Illumina's recommendations, before being sequenced on a NextSeq 500 using the high-output flow cell and the v2.5, 300-cycle chemistry. The sequencing process yielded 140 gigabases that met the Q30 threshold.

The metagenomic reads underwent processing using metaWRAP v = 1.3.2 (Uritskiy et al., 2018), following the procedures outlined in the tool's GitHub page “Useage_tutorial.md” (Accessed 10 Oct 2022). In summary, individual sample assembly was performed using metaSPAades v3.11.1 (Nurk et al., 2017) on the seven samples, including three from the ice surface, one from the biofilm, and three from cryoconite sediment. Metagenomic bins were constructed using metaBAT2 v2.12.1 (Kang et al., 2019), MaxBin2 v2.2.5 (Wu et al., 2016), and CONCOCT v1.0.0 (Wehrmann et al., 2017). The resulting bins underwent reassembly and refinement with a threshold of >90 % completeness and <5 % contamination, yielding high-quality assembled metagenomes. MAGs were identified using GTDB-Tk v 2.1.1 (Parks et al., 2018) and changed manually to match NCBI taxonomy at phylum level to most accurately compare the MAGs to the cultured isolates.

### 2.3 Nanopore whole-genome sequencing

DNA extraction was performed using a Gentra Puregene kit (Qiagen, Hilden, Germany), following the manufacturer's protocol, except for the DNA hydration solution. Instead, 10 mM Tris, 50 mM NaCl pH 8.0 was used. The quality of the DNA eluates was measured on a Nanodrop 2000 spectrophotometer (Thermo Scientific) and the quantity was measured on a Qubit4 fluorometer (Thermo Scientific) using the BR DNA assay.

Libraries for Oxford Nanopore Technologies (ONT) Sequencing were prepared using the Rapid Barcoding kit SQK-RBK110.96, following the manufacturer's instructions (Oxford Nanopore Technologies). Sequencing was performed on a MinION mk1b using a R.4.1 flowcell controlled by MinKNOW 22.10.10. Basecalling was performed using GPU-Guppy version 6.4.6+ae70e8f under default settings. The resulting fastq files were error-corrected using LorDEC (Salmela and Rivals, 2014), with the Illumina pair-end reads generated previously (Jaarsma et al., 2023).

The corrected long reads were used for *de novo* whole genome assemblies with Flye (Kolmogorov et al., 2020) under default settings utilizing the –nano-corr flag. The assembly graph files of the genomes were visualized using Bandage (Wick et al., 2015). The assembled genomes were inspected for completion using BUSCO (Simão et al., 2015) and its generalized bacteria_odb10 (2020-03-06) database, and were annotated using PROKKA (Seemann, 2014) under default settings.

The Type strain Genome Server (TYGS) was used to identify close relatives of the resulting nanopore genomes and MAGs, where possible (Meier-Kolthoff and Göker, 2019). Alternatively, full-length 16S rRNA genes were used to identify closest relatives in the NCBI database using BLASTn.

### 2.4 Genome mining workflow

#### 2.4.1 BGC detection

Biosynthetic Gene Clusters (BGCs) were detected using a local version of antiSMASH version 6.1.1 (Blin et al., 2021). No gene-finding tool was used for isolate genomes as they were already annotated. MAGs were annotated using prodigal-m. Full parameters can be found in the Github repository (https://github.com/AU-ENVS-Bioinformatics/GR21_genome_mining).

#### 2.4.2 Similarity network

BiG-SCAPE version 1.1.2 (Navarro-Muñoz et al., 2020) was used to construct a similarity network of the predicted BGCs, including singletons, together with the MIBiG dataset (Terlouw et al., 2023). Gene cluster families (GCFs) and gene cluster clans (GCCs) were created using the default 0.30 and 0.70 cutoff values, respectively.

### 2.5 Data handling and visualization

AntiSMASH and BiG-SCAPE output data were parsed using a custom R script (https://github.com/AU-ENVS-Bioinformatics/RauENVS/blob/main/R/parse_antismash.R). One BGC classified as Saccharide was moved to the category “Other”, due to it being the only Saccharide detected.

The similarity network was created using Cytoscape version 3.9.1 (Shannon et al., 2003). Gene cluster comparison figures were constructed using Clinker (Gilchrist and Chooi, 2021).

Drep was used to calculate MASH ANI (Olm et al., 2017).

R scripts used to plot boxplots, bar plots and Venn diagram can be found in our Github repository (https://github.com/AU-ENVS-Bioinformatics/GR21_genome_mining).

## 3 Results

### 3.1 The biosynthetic potential of Greenland Ice Sheet genomes

The collection of mined genomes included 133 high-quality MAGs and 28 genomes from bacterial isolates. Most MAGs originated from the cryoconite metagenomes (89), followed by the ice (32) and biofilm (12) metagenomes (Jaarsma et al., 2023). Isolates originated predominantly from cryoconite (13) and ice (10), but with few from the biofilm (3) and the snow (2). It was found that these 28 isolates are a less diverse group of organisms that is less abundant in the environment compared to the MAGs. Furthermore, the isolates and MAGs contain little genetic overlap, despite them originating from the same environment (Jaarsma et al., 2023).

After nanopore sequencing complete genomes were obtained for all except three of the 28 genomes, the exceptions being almost complete but not closed. Only eight genomes could be identified to species level with valid DNA-DNA Hybridization (DDH) and GC values using TYGS. This indicates that the genome collection contains many potentially new species. A full table of the genome collection including environment, closest relatives based on TYGS and the NCBI 16S rRNA database, genome size, GC content, number of genes, plasmids, rRNA operons and tRNA genes can be found in Supplementary material S1.

A total of 848 Biosynthetic Gene Clusters (BGCs) were obtained, 641 from MAGs and 207 from isolate genomes. Only 25 % of BGCs obtained from the MAGs were found to be complete (i.e., not located on a contig edge). For this reason, “glocal” alignment mode was used in BiG-SCAPE, accounting for potential fragmented BGCs. One BGC, encoding a nonribosomal peptide synthetase (NRPS) was found to be located on a plasmid, in an ice surface *Herbaspirillum* isolate (5I1). Out of the 71 types of BGC that can be recognized by antiSMASH, 33 were obtained in total, with 25 types in the isolate genomes and 23 types in the MAGs (Supplementary material S2).

**Figure 2:**
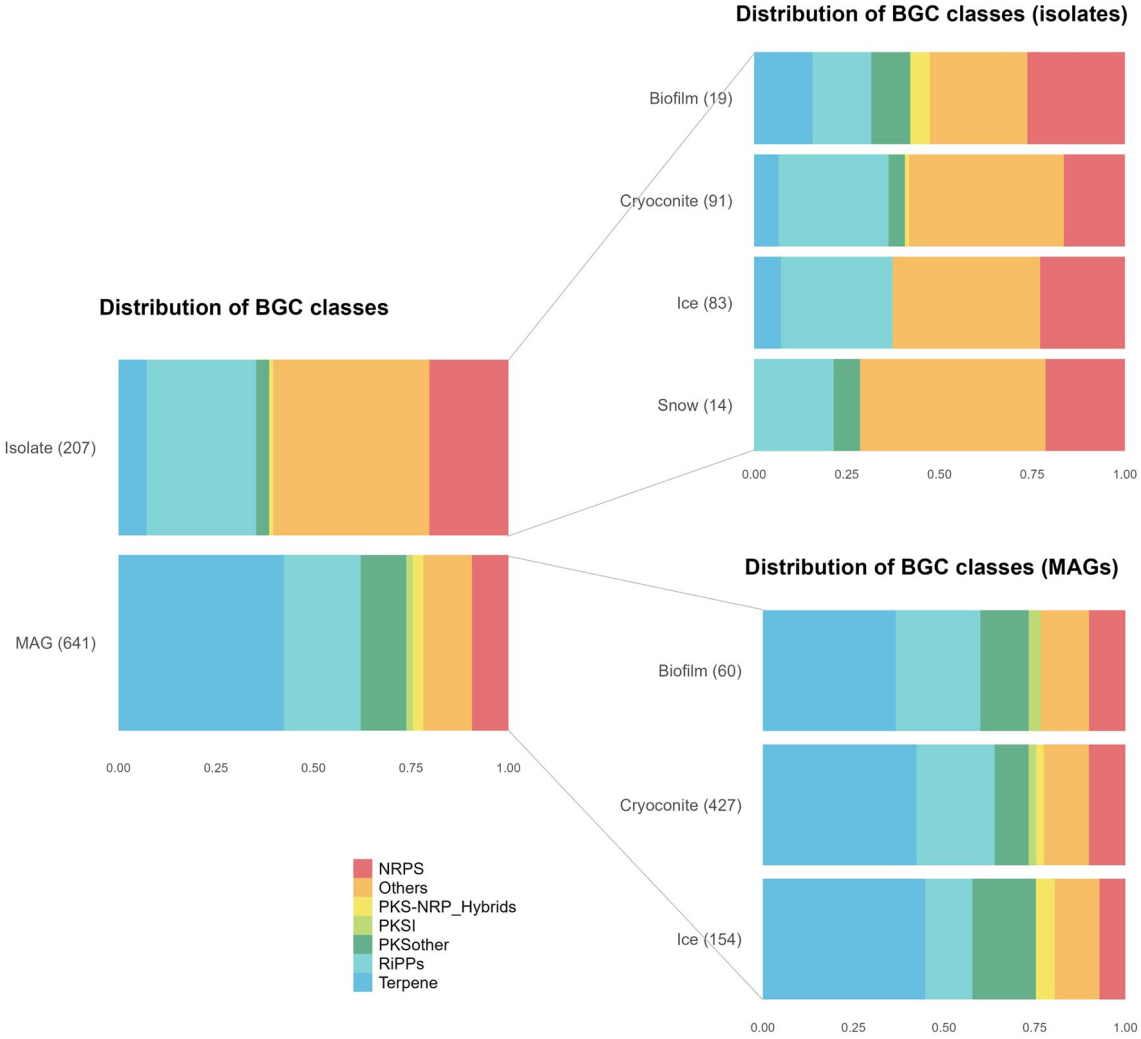
Distribution of Biosynthetic Gene Cluster (BGC) classes in metagenome-assembled genomes (MAGs) and isolate genomes, divided into environments. NRPS, nonribosomal peptide synthetase; NRP, nonribosomal peptide; PKS, polyketide synthase; PKSI, type I polyketide synthase; RiPPs, ribosomally synthesized and post-translationally modified peptides.

The distribution of BGC classes, as assigned by BiG-SCAPE, differed between isolate genomes and MAGs, the most notable difference being a much larger abundance of terpenes among the MAG BGCs (42 vs. 7 % of BGCs) (Figure 2). The distribution of MAG BGCs is similar among the different environments, while this distribution is more different for the isolate BGCs. Notably, no terpene BGCs were found among snow BGCs.

**Figure 3:**
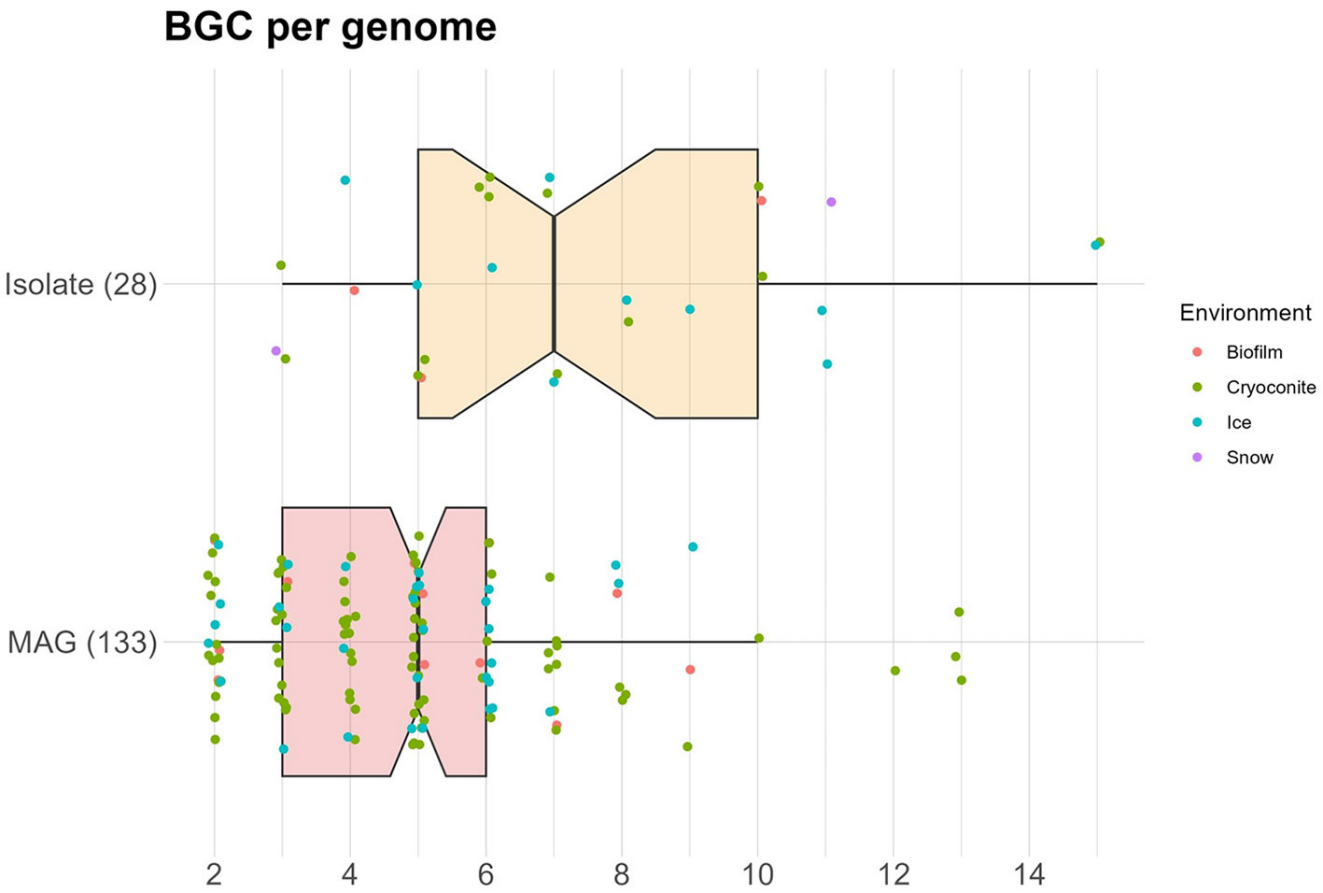
Notched box plot showing the number of BGCs per genome, colored by environment. Number of genomes is given in parentheses.

**Figure 4:**
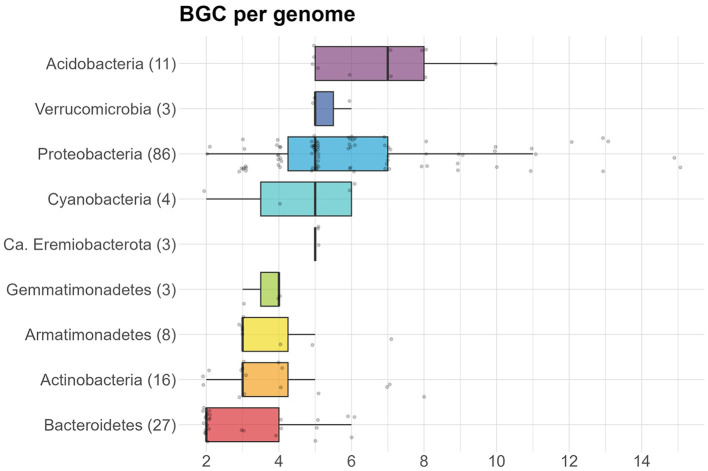
Box plot showing the number of BGCs per genome, separated by phylum. Number of genomes is given in parentheses for each phylum.

On average, 5.3 BGCs were predicted per genome, with a mode of 5. The average was higher for the isolates (7.3) than for the MAGs (4.8). In addition, the median number of predicted BGCs per genome was significantly higher for the isolates (7) compared to the MAGs (5) (Wilcoxon Rank Sum test, *p*−*value* < 0.0001) (Figure 3). Organisms with a high number of BGCs are often referred to as “talented”. Here, we define a producer as talented when it contains a number of BGCs that is equal to or over twice the median value. Talented producers were found among isolates as well as MAGs. When investigating the number of BGCs per genome for each phylum separately, the highest median was found in Acidobacteria (7), with the entire interquartile range (IQR) being equal or higher to the overall median (Figure 4). The lowest median number of BGCs per genome was found in Bacteroidetes (2), and the entire IQRs of Bacteroidetes, Actinobacteria, Armatimonadetes, and Gemmatimonadetes were lower than the overall median. The most talented producers were found among the Proteobacteria, with up to 15 BGCs per genome (Supplementary material S3).

### 3.2 Similarity network analysis of obtained biosynthetic diversity

**Figure 5:**
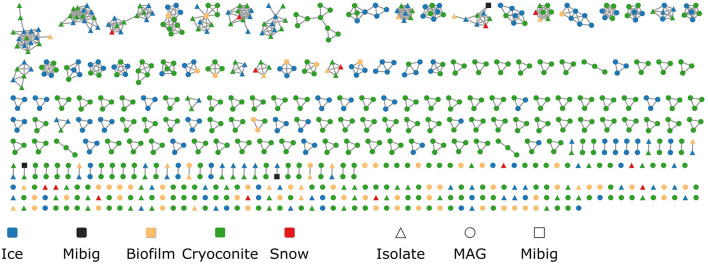
Similarity network of obtained BGCs. Each point represents a BGC, colored by environment. Shapes indicate if the BGC was found in a MAG or isolate genome. Matches to the MIBiG database are shown as black squares.

Using BiG-SCAPE, the Biosynthetic Gene Clusters (BGCs) were organized into 410 Gene Cluster Families (GCFs), the biggest containing 13 BGCs. Only 215 BGCs remained as singletons (Figure 5). While 307 GCFs were unique to MAGs, 98 GCFs were unique to isolate genomes. Only five GCFs were obtained from both MAGs and isolate genomes. Cryoconite genomes harbored the most unique GCFs (217), followed by ice (66), biofilm (57), and snow (8). No GCFs were obtained that included BGCs from all four environments. The biggest overlap was found between ice and cryoconite, with 46 shared GCFs. A Venn diagram of GCFs from the different environments can be found in Supplementary material S4. Genomes from cryoconite harbored the largest proportion of unique GCFs (81 %), followed by genomes from biofilm (75 %). Genomes from snow and ice contained a smaller proportion of unique GCFs, with 57 and 54 %, respectively. It is important to note that these results can be influenced by the number of samples analyzed for each habitat.

**Figure 6:**
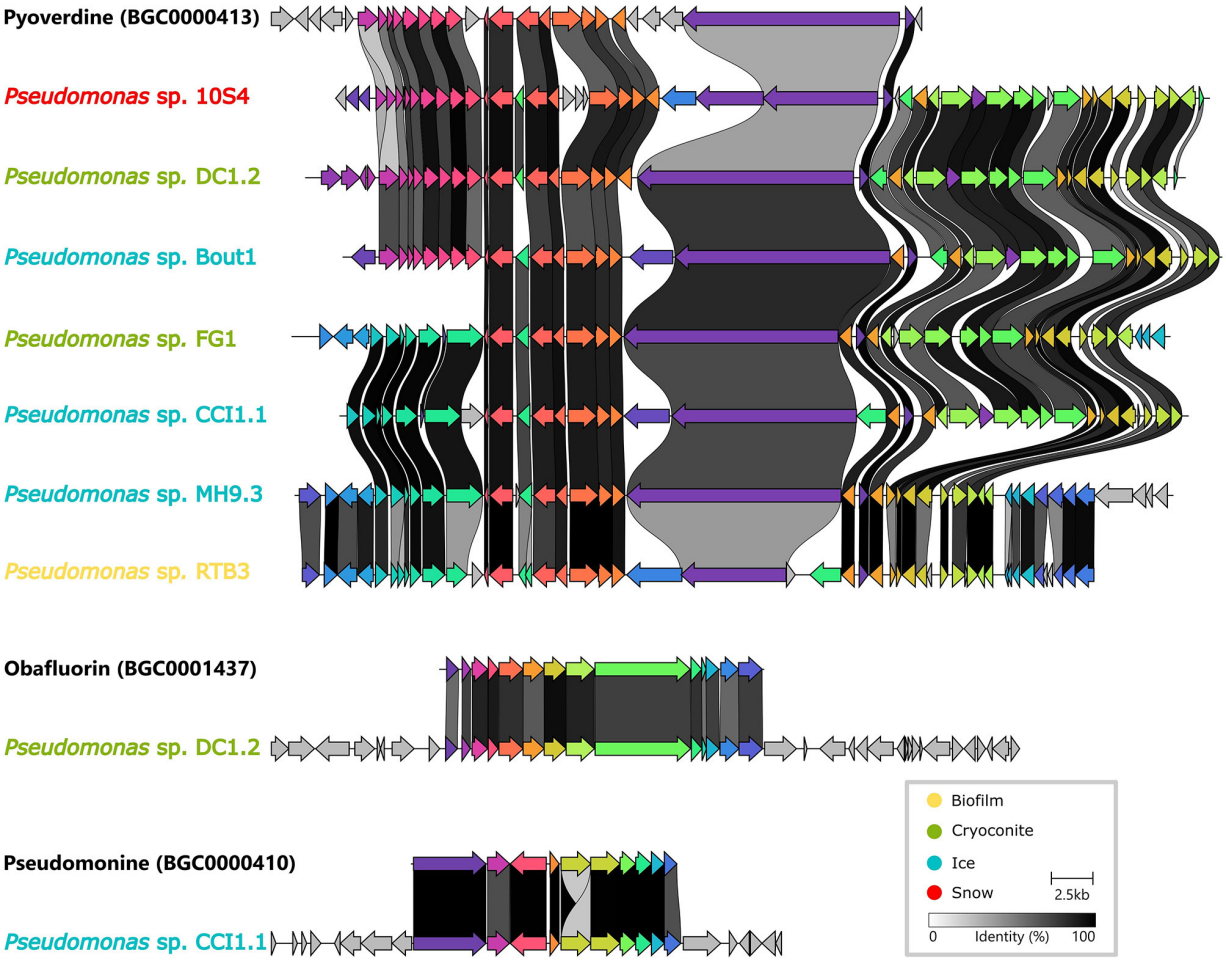
Pairwise alignment of genes (arrows) from isolate Biosynthetic Gene Clusters (BGCs) with genes from three homologous BGCs from the MIBiG repository (names in black).

It was investigated whether homologous Biosynthetic Gene Clusters have previously been reported by comparing our dataset to the MIBiG repository. Three Gene Cluster Families (GCFs) contained a MIBiG reference BGC (Figure 6). Firstly, a Non-Ribosomal Peptide (NRP) BGC from *Pseudomonas protegens* PF5, encoding pyoverdine, a well-described siderophore (Stintzi et al., 1999) (BGC0000413), was part of a Gene Cluster Clan (GCC) containing two GCFs (2078 and 2111). This clan, with seven BGCs from *Pseudomonas* isolates, was the only GCC containing BGCs from all four environments. In addition, two more MIBiG reference BGCs from *Pseudomonas fluorescens* clustered with two isolate BGCs. Firstly, a BGC encoding obafluorin, which is a β-lactone antibiotic (Schaffer et al., 2017), (BGC0001437) was found in the same GCF (1404) as a BGC from an isolate from cryoconite. Secondly, a pseudomonine BGC (BGC0000410), encoding a siderophore (Mercado-Blanco et al., 2001), was part of a GCF (449) together with a BGC from an ice sample isolate. The latter two isolates are *Pseudomonas* species, and both also contain a BGC that was part of the aforementioned pyoverdine GCF.

**Figure 7:**
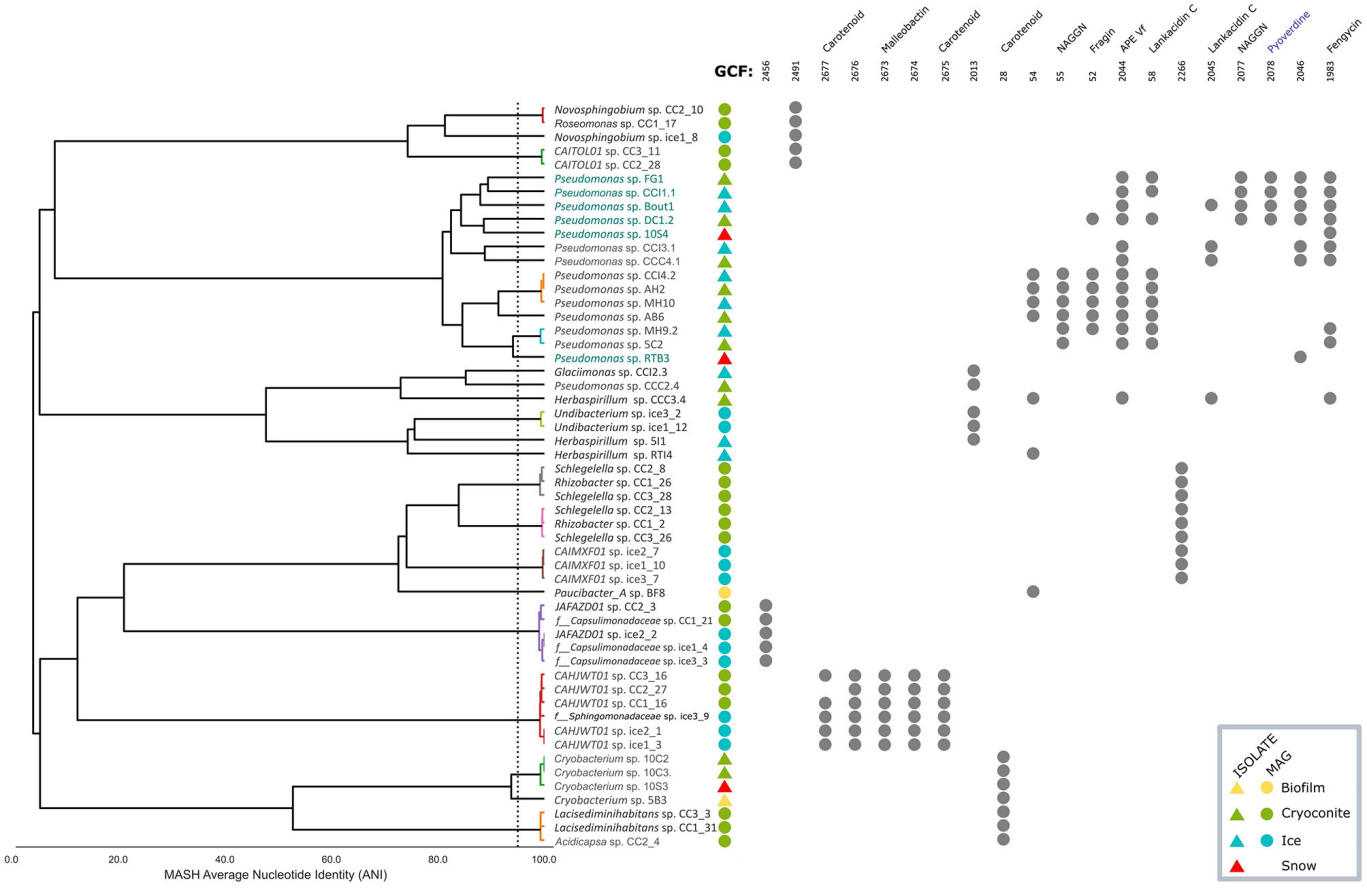
Presence/absence plot of the 20 most abundant GCFs, with average nucleotide identity (ANI) clustering of 54 genomes of their producers. The dashed line indicates an ANI of 95 %. Producers with a name in teal are considered talented with 10 or more BGCs encoded. Colored polygons indicate sample origin of the genomes. Where possible, names of known BGCs with similarity are given for each GCF. Pyoverdine, shown in blue, is a MiBIG reference BGC that was placed in a GCF with isolate genomes by BiGSCAPE (see also Figure 6).

A presence/absence plot was made of the 20 most abundant GCFs, also showing the average nucleotide identity (ANI) clustering of the 54 genomes that harbor these 20 GCFs (Figure 7). This group of genomes contained six talented producers that contained 10 or more BGCs. Where possible, similar BGCs, as predicted by antiSMASH, are noted for each GCF. Three GCFs obtained only from MAGs were related to carotenoids, with one BGC in GCF 2677 having 66 % similarity to a zeaxanthin BGC from *Xanthobacter autotrophicus Py2* (BGC0000656). The similarity of the five identified BGCs in GCF 2677 was limited to one gene, *crtB*, which encodes a phytoene synthase, which makes a colorless precursor of the carotenoid zeaxanthin (Larsen et al., 2002). In addition, the identified BGCs contained up to five other genes, potentially encoding the full biosynthesis of a carotenoid pigment (Supplementary material S5).

Two GCFs (55 and 2,077) were of the N-acetylglutaminylglutamine amide (NAGGN) type, a compatible solute (Kurz et al., 2010). One GCF (2044) is similar (40 %) to an aryl polyene (APE) BGC from *Aliivibrio fischeri ES114* (BGC0000837). Being attached to the outer membrane in Gram-negative bacteria, APEs are thought to enable interactions from the host with its environment, such as biofilm formation (Johnston et al., 2021). Another GCF (52) was 37 % similar to a fragin BGC (BGC0001599), which has antifungal activity (Jenul et al., 2018). One GCF (1983) showed 20 % similarity to a fengycin BGC (BGC0001095), encoding a lipopetide with antifungal activity (Vanittanakom et al., 1986). Two GCFs (58 and 2045) showed 13 % similarity to a lankacidin C BGC (BGC0001100), which has antimicrobial activity (Cai et al., 2019). Low similarity (7 %) was observed between one GCF (2673) and a malleobactin A BGC (BGC0000386), encoding a siderophore (Franke et al., 2014).

In accordance with the distribution of the entire collection of GCFs, there was little overlap between the 20 most abundant GCFs from MAGs and isolate genomes. One carotenoid GCF (28) was, however, observed in MAGs and isolate genomes, as well as two GCFs with unknown function (2013 and 54). The latter GCF 54 contained BGCs from genomes in separate clusters in the ANI tree.

## 4 Discussion

In this study, an extensive set of predicted Biosynthetic Gene Clusters (BGCs) was obtained from a collection of metagenomes and cultured isolate genomes obtained from supraglacial habitats on the Greenland Ice Sheet. The distribution of these BGCs revealed that isolates had a significantly higher median of BGCs per genome compared to the MAGs. Furthermore, few Gene Cluster Families were shared between multiple sampled environments. We found evidence for the capacity to produce natural products that might aid in adaptation to life in the cryosphere. The majority of BGCs were however of thus far unknown function, highlighting the large potential of supraglacial bacterial genomes for bioprospecting.

### 4.1 Distribution of BGCs within habitats and genomes

A significantly higher median number of BGCs per genome was found for isolates compared to MAGs. Acidobacteria had the highest median number of BGCs per genome, but the Proteobacteria yielded the most talented producers. On average, more BGCs were observed per genome for both the MAGs (4.8 BGCs/genome) and isolates (7.3 BGCs/genome) in our collection than was observed in a large genome mining study of over 170,000 publicly available isolates (6.2 BGCs/genome) and over 47,000 MAGs (2.7 BGCs/genome) (Gavriilidou et al., 2022).

Very little overlap in biosynthetic potential was observed between isolates and MAGs: only five out of 410 identified Gene Cluster Families (GCFs) were shared between the two groups. This is in line with the observation that the isolates and MAGs contain little genetic overlap. Similarly, little overlap was found in the biosynthetic potential coming from the different environments. No GCFs were shared between all four environments, although this could be explained by sample size differences. In our study, it is challenging to ensure equal representation of various habitats due to differences in sample types. Efforts were made to maximize biodiversity captured in ice surface and cryoconite habitats by combining multiple subsamples for deep metagenomic sequencing. However, this approach was not feasible for the more unique snow and biofilm samples. While the biofilm was used for metagenome sequencing, the snow habitat remains under-sampled as only isolates were included. Each environment had unique GCFs, possibly encoded by specialist organisms from those specific environments. Conversely, there were also some overlapping GCFs, especially between ice and cryoconite. Microbes living in distinct environments may have gained the same functionalities; for instance, there are GCFs present among different genera (Figure 7). This could reflect horizontal gene transfer, a common way of transfer of biosynthetic gene clusters (Fischbach et al., 2008). Alternatively, it could be that the same organism is simply found in multiple environments.

In only three GCFs did BGCs (exclusively from isolates) cluster together with a MIBiG reference BGC. The minimal similarity with MIBiG BGCs is not surprising, as this database is mainly based on cultured isolates. Even though the collection in this study contained a number of isolate genomes, they are likely understudied representatives. A similar observation was made in the analysis of MAGs from activated sludge microorganisms, where none of the predicted BGCs clustered into GCFs with MIBiG BGCs (Sánchez-Navarro et al., 2022). Furthermore, it has been estimated that only 3 % of the potential encoded bacterial encoded natural products have been characterized experimentally (Gavriilidou et al., 2022). Many novel BGCs were also discovered in 3,241 genomes, including MAGs and isolates, from Tibetan glaciers (Liu et al., 2022). Similarly to the Tibetan glaciers, terpenes were the most abundant BGCs and most BGCs were obtained from Proteobacteria from the Greenland Ice Sheet. However, while Myxococcota contained the highest number of BGCs per genome within the Tibetan glaciers, they were not present in our genome collection.

### 4.2 Biosynthetic potential reflects environmental adaptation

While many of the predicted BGCs were of unknown function, those that could be linked to similar BGCs with a known function included BGCs encoding antimicrobials, siderophores, compatible solutes, and carotenoid pigments. It has previously been found that volatile organic compounds emitted from bare ice surfaces on the Greenland Ice Sheet include compounds with a reported antifungal activity (Doting et al., 2022). In addition, the pigment produced by glacier ice algae, purpurogallin carboxylic acid-6-O-β-D- glucopyranoside, has been suggested to have an antimicrobial activity next to its photoprotective activity (Remias et al., 2012). Our evidence for the capability to produce antimicrobial natural products adds to the indications for a potential role of natural products in microbial interactions in supraglacial habitats of the Greenland Ice Sheet.

Furthermore, we found BGCs that might be involved in adaptation for life in the cryosphere. Carotenoid pigments offer protection against harmful UV radiation (Krinsky, 1978), and it is therefore not surprising that glacial microorganisms use pigments as sunscreen to protect against the harsh sunlight that is often found in high latitudes and altitudes. Carotenoid pigments are also found to regulate membrane fluidity in response to temperature fluctuations (De Maayer et al., 2014). Carotenoid pigments have for instance been identified in bacteria from Antarctica (Dieser et al., 2010; Vila et al., 2019). Carotenoid pigments are also present in the pigment pool of supraglacial communities in Greenland (Halbach et al., 2022), and in the metabolome of the Foxfonna ice cap in Svalbard (Gokul et al., 2023). Liu et al. (2022) have furthermore speculated that the large number of terpene BGCs in Tibetan glacier genomes may be explained by the presence of carotenoid pigments in bacteria.

It has been suggested that pigments in eukaryotic glacier algae not only help shield against UV radiation, but may also play a role in the production of liquid water by accelerating the melt of the local frozen environment (Dial et al., 2018). If the same mechanism also occurs in pigmented bacterial cells is yet to be tested, but in theory it would increase the cell's fitness if it was able to ensure a film of liquid water around itself, especially for those living on the ice surface. It has been speculated that surface microbes other than eukaryotic algae could also play a role in biological albedo reduction (Hotaling et al., 2021). It is therefore worthwhile investigating the extent of bacterial pigmentation on the ice surface further. In addition, evidence for the biosynthesis capacity of the compatible solute NAGGN was found among the ubiquitous GCFs. The accumulation of compatible solutes like NAGGN is linked to protection against freezing and osmotic stress (D'Souza-Ault et al., 1993; De Maayer et al., 2014). Another stressor that is associated with cold environments is low nutrient concentrations (De Maayer et al., 2014), and this may be reflected in the biosynthetic potential of supraglacial bacteria. While it has not yet been truly tested whether the concentration of iron on the surface of the Greenland Ice Sheet is limiting microbial growth, the presence of BGCs encoding siderophores suggests a role for these natural products in scavenging iron as a potential environmental adaptation.

It seems, therefore, that Greenland Ice Sheet microbes are equipped with biosynthetic potential to help survive the extreme conditions of their habitat, either through physical adaptations or through inhibition of the growth of their competitors using antimicrobial compounds. However, the presence of the biosynthetic capacity to produce above mentioned natural products does not necessarily mean that these natural products are actually being produced in supraglacial habitats. The ecological function of many BGCs therefore remains speculative. For instance, achieving sufficient concentrations for extracellularly-active antimicrobial natural products to function is challenging, especially on the constantly diluted melting ice surface. According to the Screening Hypothesis, evolution favors organisms with a large capacity to produce a wide chemical diversity. Rather than the selective pressure acting on individual BGCs, the pressure therefore acts on the biosynthetic capacity itself, and as a consequence, not every natural product will have a biological function (Firn and Jones, 2003). A transcriptomics approach could be used to investigate whether the BGCs identified here are actually transcribed *in situ* on the ice sheet.

### 4.3 Implications for bioprospecting

The majority of BGCs identified in this study, including those in several of the most ubiquitous GCFs, were of unknown function, encoding potentially unknown chemistry. This unknown chemistry may include novel antimicrobials and other biotechnologically relevant compounds. This illustrates the high potential for bioprospecting of these under-explored organisms, but also the large need for functional characterization of many of these BGCs, including screening for activity under lab conditions.

Metagenome-assembled genomes and isolates offer distinct benefits. Isolates, although more easily cultivable, are often inadequately represented in the environment. Conversely, MAGs exhibit stronger representation, yet they frequently remain uncultured. Isolates had a significantly higher median of BGCs per genome compared to MAGs. About 25 % of GCFs in this study were found in cultured isolates, allowing relatively more straightforward characterization of their encoded natural products. The remainder could be explored through heterologous expression, for instance in a cold-adapted expression hosts such as *Aliivibrio wodanis* (Söderberg et al., 2019), *Shewanella livingstonensis* (Kawai et al., 2019), or the yeast *Debaromyces macquariensis* (Wanarska et al., 2022). Many BGCs from MAGs were found to be on contig edges, despite the MAGs being of high quality. It has previously been found that high-quality MAGs assembled from long reads yield more complete BGCs compared to those assembled from short reads (Sánchez-Navarro et al., 2022), highlighting the benefits of long read-assembled high-quality MAGs for future genome mining studies.

In accordance with the low genetic overlap between the isolates and MAGs, there is also little overlap in biosynthetic potential. By harnessing both metagenomic and cultured sources, the prospects of discovering novel compounds are enhanced by tapping into complementary genetic and chemical diversity.

In conclusion, this study identified a diverse set of biosynthetic gene clusters (BGCs) in genomes from the Greenland Ice Sheet, revealing potential survival strategies and substantial untapped resources for bioprospecting in this extreme environment.

## Data availability statement

The datasets presented in this study can be found in online repositories. The names of the repository/repositories and accession number(s) can be found below: https://www.ncbi.nlm.nih.gov/, PRJNA942590.

## Author contributions

AJ: Conceptualization, Data curation, Formal analysis, Investigation, Methodology, Software, Writing—original draft, Writing—review & editing. AZ: Conceptualization, Data curation, Formal analysis, Methodology, Software, Writing—review & editing. KS: Conceptualization, Data curation, Formal analysis, Methodology, Software, Writing—review & editing. FC: Data curation, Methodology, Software, Writing—review & editing. AS: Conceptualization, Formal analysis, Investigation, Methodology, Writing—review & editing. LS: Conceptualization, Formal analysis, Investigation, Methodology, Writing—review & editing. MST: Conceptualization, Methodology, Writing—review & editing. PS: Conceptualization, Methodology, Writing—review & editing. LB: Funding acquisition, Project administration, Writing—review & editing. MT: Funding acquisition, Project administration, Writing—review & editing. AA: Conceptualization, Funding acquisition, Methodology, Project administration, Writing—review & editing.

## References

[B1] Anesio A. M. Lutz S. Chrismas N. A. Benning L. G. (2017). The microbiome of glaciers and ice sheets. NPJ Biofilms Microbio. 3, 0–1. 10.1038/s41522-017-0019-0 28649411 PMC5460203

[B2] Athanasopoulou K. Boti M. A. Adamopoulos P. G. Skourou P. C. Scorilas A. (2022). Third-generation sequencing: the spearhead towards the radical transformation of modern genomics. Life 12, 30. 10.3390/life12010030 35054423 PMC8780579

[B3] Blin K. Shaw S. Kloosterman A. M. Charlop-Powers Z. Van Wezel G. P. Medema M. H.. (2021). AntiSMASH 6.0: Improving cluster detection and comparison capabilities. Nucleic Acids Res. 49, W29–W35. 10.1093/nar/gkab335 33978755 PMC8262755

[B4] Bourquin M. Busi S. B. Fodelianakis S. Peter H. Washburne A. Kohler T. J.. (2022). The microbiome of cryospheric ecosystems. Nat. Commun. 13, 1–9. 10.1038/s41467-022-30816-4 35655063 PMC9163120

[B5] Cai X. Zhao L. Bode H. B. (2019). Reprogramming promiscuous nonribosomal peptide synthetases for production of specific peptides. Org. Lett. 21, 2116–2120. 10.1021/acs.orglett.9b00395 30859835

[B6] Cook J. M. Tedstone A. J. Williamson C. McCutcheon J. Hodson A. J. Dayal A.. (2020). Glacier algae accelerate melt rates on the south-western Greenland Ice Sheet. Cryosphere 14, 309–330. 10.5194/tc-14-309-2020

[B7] Cordier C. Morton D. Murrison S. Nelson A. O'Leary-Steele C. (2008). Natural products as an inspiration in the diversity-oriented synthesis of bioactive compound libraries. Nat. Prod. Rep. 25, 719–737. 10.1039/b706296f 18663392 PMC2496956

[B8] Cragg G. M. Newman D. J. (2013). Natural products: a continuing source of novel drug leads. Biochimica et Biophysica Acta - General Subjects 1830, 3670–3695. 10.1016/j.bbagen.2013.02.008 23428572 PMC3672862

[B9] De Maayer P. Anderson D. Cary C. Cowan D. A. (2014). Some like it cold: understanding the survival strategies of psychrophiles. EMBO Rep. 15, 508–517. 10.1002/embr.201338170 24671034 PMC4210084

[B10] Dial R. J. Ganey G. Q. Skiles S. M. K. (2018). What color should glacier algae be? An ecological role for red carbon in the cryosphere. FEMS Microbiol. Ecol. 94, 7. 10.1093/femsec/fiy007 29346532

[B11] Dieser M. Greenwood M. Foreman C. M. (2010). Carotenoid pigmentation in Antarctic heterotrophic bacteria as a strategy to withstand environmental stresses. Arct. Antarct. Alp. Res. 42, 396–405. 10.1657/1938-4246-42.4.396

[B12] Doting E. L. Davie-Martin C. L. Johansen A. Benning L. G. Tranter M. Rinnan R.. (2022). Greenland ice sheet surfaces colonized by microbial communities emit volatile organic compounds. Front. Microbiol. 13, 1–14. 10.3389/fmicb.2022.886293 35747370 PMC9211068

[B13] D'Souza-Ault M. R. Smith L. T. Smith G. M. (1993). Roles of N-acetylglutaminylglutamine amide and glycine betaine in adaptation of Pseudomonas aeruginosa to osmotic stress. Appl. Environ. Microbiol. 59, 473–478. 10.1128/aem.59.2.473-478.1993 8434912 PMC202129

[B14] Feng S. Cook J. M. Anesio A. M. Benning L. G. Tranter M. (2023). Long time series (1984-2020) of albedo variations on the Greenland ice sheet from harmonized Landsat and Sentinel 2 imagery. J. Glaciol. 6, 1–16. 10.1017/jog.2023.11

[B15] Firn R. D. Jones C. G. (2003). Natural products - a simple model to explain chemical diversity. Nat. Prod. Rep. 20, 382–391. 10.1039/b208815k 12964834

[B16] Fischbach M. A. Walsh C. T. Clardy J. (2008). The evolution of gene collectives: How natural selection drives chemical innovation. Proc. Natl. Acad. Sci. USA. 105, 4601–4608. 10.1073/pnas.0709132105 18216259 PMC2290807

[B17] Franke J. Ishida K. Hertweck C. (2014). Evolution of siderophore pathways in human pathogenic bacteria. J. Am. Chem. Soc. 136, 5599–5602. 10.1021/ja501597w 24707815

[B18] Gavriilidou A. Kautsar S. A. Zaburannyi N. Krug D. Müller R. Medema M. H.. (2022). Compendium of specialized metabolite biosynthetic diversity encoded in bacterial genomes. Nat. Microbiol. 7, 726–735. 10.1038/s41564-022-01110-2 35505244

[B19] Ghoul M. Mitri S. (2016). The ecology and evolution of microbial competition. Trends Microbiol. 24, 833–845. 10.1016/j.tim.2016.06.011 27546832

[B20] Gilchrist C. L. Chooi Y. H. (2021). Clinker & clustermap.js: automatic generation of gene cluster comparison figures. Bioinformatics 37, 2473–2475. 10.1093/bioinformatics/btab007 33459763

[B21] Gokul J. K. Mur L. A. J. Andrew A. Hodson J. Tristram Irvine-Fynn D. L.. (2023). Icescape-scale metabolomics reveals cyanobacterial and topographic control of the core metabolism of the cryoconite ecosystem of an Arctic ice cap. Environm. Microbiol. 25, 2549–2563. 10.1111/1462-2920.16485 37621052

[B22] Halbach L. Chevrollier L. A. Doting E. L. Cook J. M. Jensen M. B. Benning L. G.. (2022). Pigment signatures of algal communities and their implications for glacier surface darkening. Sci. Rep. 12, 1–14. 10.1038/s41598-022-22271-4 36271236 PMC9587043

[B23] Hotaling S. Lutz S. Dial R. J. Anesio A. M. Benning L. G. Fountain A. G.. (2021). Biological albedo reduction on ice sheets, glaciers, and snowfields. Earth-Sci. Rev. 220, 103728. 10.1016/j.earscirev.2021.103728

[B24] Hug L. A. Baker B. J. Anantharaman K. Brown C. T. Probst A. J. Castelle C. J.. (2016). A new view of the tree of life. Nat. Microbiol. 1, 48. 10.1038/nmicrobiol.2016.48 27572647

[B25] Jaarsma A. H. Sipes K. Zervas A. Jiménez F. C. Ellegaard-Jensen L. Thogersen M. S.. (2023). Exploring microbial diversity in Greenland Ice Sheet supraglacial habitats through culturing-dependent and -independent approaches. FEMS Microbiol. Ecol. 99, fiad119. 10.1093/femsec/fiad119 37791411 PMC10580271

[B26] Jenul C. Sieber S. Daeppen C. Mathew A. Lardi M. Pessi G.. (2018). Biosynthesis of fragin is controlled by a novel quorum sensing signal. Nat. Commun. 9, 2. 10.1038/s41467-018-03690-2 29602945 PMC5878181

[B27] Johnston I. Osborn L. J. Markley R. L. McManus E. A. Kadam A. Schultz K. B.. (2021). Identification of essential genes for *Escherichia coli* aryl polyene biosynthesis and function in biofilm formation. NPJ Biofilms Microbio. 7, 3. 10.1038/s41522-021-00226-3 34215744 PMC8253772

[B28] Kang D. D. Li F. Kirton E. Thomas A. Egan R. An H.. (2019). MetaBAT 2: An adaptive binning algorithm for robust and efficient genome reconstruction from metagenome assemblies. PeerJ 2019(7). 10.7717/peerj.7359 31388474 PMC6662567

[B29] Kawai S. Kawamoto J. Ogawa T. Kurihara T. (2019). Development of a regulatable low-temperature protein expression system using the psychrotrophic bacterium, Shewanella livingstonensis Ac10, as the host. Biosci. Biotechnol. Biochem. 83, 2153–2162. 10.1080/09168451.2019.1638754 31291825

[B30] Kolmogorov M. Bickhart D. M. Behsaz B. Gurevich A. Rayko M. Shin S. B.. (2020). metaFlye: scalable long-read metagenome assembly using repeat graphs. Nat. Methods 17, 1103–1110. 10.1038/s41592-020-00971-x 33020656 PMC10699202

[B31] Krinsky N. I. (1978). Non-photosynthetic functions of carotenoids. Philos. Trans. R. Soc. Lond. B Biol. Sci. 284, 581–590. 10.1098/rstb.1978.0091

[B32] Kurz M. Buren A. Y. Seip B. Lindow S. E. Gross H. (2010). Genome-driven investigation of compatible solute biosynthesis pathways of *Pseudomonas syringae* pv. syringae and their contribution to water stress tolerance. Appl. Environm. Microbiol. 76, 5452–5462. 10.1128/AEM.00686-10 20581190 PMC2918956

[B33] Larsen R. A. Wilson M. M. Guss A. M. Metcalf W. W. (2002). Genetic analysis of pigment biosynthesis in *Xanthobacter autotrophicus* Py2 using a new, highly efficient transposon mutagenesis system that is functional in a wide variety of bacteria. Arch. Microbiol. 178:193–201. 10.1007/s00203-002-0442-2 12189420

[B34] Linde K. V. D. Lim B. T. Rondeel J. M. M. Antonissen L. P. M. T. Jong G. M. T. D. (2000). Improved bacteriological surveillance of haemodialysis fluids : a comparison between Tryptic soy agar and Reasoners 2A media. Nephrol. Dialysis Transplant. 1999, 2433–2437. 10.1093/ndt/14.10.2433 10528669

[B35] Liu Y. Ji M. Yu T. Zaugg J. Anesio A. M. Zhang Z.. (2022). A genome and gene catalog of glacier microbiomes. Nat. Biotechnol. 40, 1341–1348. 10.1038/s41587-022-01367-2 35760913

[B36] Lutz S. McCutcheon J. McQuaid J. B. Benning L. G. (2018). The diversity of ice algal communities on the Greenland Ice Sheet as revealed by oligotyping. Microbial genomics 4, 159. 10.1099/mgen.0.000159 29547098 PMC5885011

[B37] Ma J. Gu Y. Xu P. (2020). A roadmap to engineering antiviral natural products synthesis in microbes. Curr. Opin. Biotechnol. 66, 140–149. 10.1016/j.copbio.2020.07.008 32795662 PMC7419324

[B38] Marcolefas E. Leung T. Okshevsky M. McKay G. Hignett E. Hamel J.. (2019). Culture-dependent bioprospecting of bacterial isolates from the canadian high arctic displaying antibacterial activity. Front. Microbiol. 10, 1836. 10.3389/fmicb.2019.01836 31447822 PMC6696727

[B39] Medema M. H. de Rond T. Moore B. S. (2021). Mining genomes to illuminate the specialized chemistry of life. Nat. Rev. Genet. 22, 553–571. 10.1038/s41576-021-00363-7 34083778 PMC8364890

[B40] Meier-Kolthoff J. P. Göker M. (2019). TYGS is an automated high-throughput platform for state-of-the-art genome-based taxonomy. Nat. Commun. 10, 3. 10.1038/s41467-019-10210-3 31097708 PMC6522516

[B41] Mercado-Blanco J. Van Der Drift K. M. Olsson P. E. Thomas-Oates J. E. Van Loon L. C. Bakker P. A. (2001). Analysis of the pmsCEAB gene cluster involved in biosynthesis of salicylic acid and the siderophore pseudomonine in the biocontrol strain *Pseudomonas fluorescens* WCS374. J. Bacteriol. 183, 1909–1920. 10.1128/JB.183.6.1909-1920.2001 11222588 PMC95085

[B42] Murray C. J. Ikuta K. S. Sharara F. Swetschinski L. Robles Aguilar G. Gray A.. (2022). Global burden of bacterial antimicrobial resistance in 2019: a systematic analysis. Lancet 399, 629–655. 10.1016/S0140-6736(21)02724-0 35065702 PMC8841637

[B43] Navarro-Mu noz J. C. Selem-Mojica N. Mullowney M. W. Kautsar S. A. Tryon J. H. Parkinson E. I.. (2020). A computational framework to explore large-scale biosynthetic diversity. Nat. Chem. Biol. 16, 60–68. 10.1038/s41589-019-0400-9 31768033 PMC6917865

[B44] Nayfach S. Roux S. Seshadri R. Udwary D. Varghese N. Schulz F.. (2020). A genomic catalog of Earth's microbiomes. Nat. Biotechnol. 39, 499–509. 10.1038/s41587-020-0718-6 33169036 PMC8041624

[B45] Nurk S. Meleshko D. Korobeynikov A. Pevzner P. A. (2017). metaSPAdes: a new versatile metagenomic assembler. Genome Res. 27, 824–834. 10.1101/gr.213959.116 28298430 PMC5411777

[B46] Olm M. R. Brown C. T. Brooks B. Banfield J. F. (2017). DRep: A tool for fast and accurate genomic comparisons that enables improved genome recovery from metagenomes through de-replication. Isme. J 11, 2864–2868. 10.1038/ismej.2017.126 28742071 PMC5702732

[B47] Parks D. H. Chuvochina M. Waite D. W. Rinke C. Skarshewski A. Chaumeil P. A.. (2018). A standardized bacterial taxonomy based on genome phylogeny substantially revises the tree of life. Nat. Biotechnol. 36, 996. 10.1038/nbt.4229 30148503

[B48] Perini L. Gostinvcar C. Likar M. Frisvad J. C. Kostanjvsek R. Nicholes M.. (2022). Interactions of fungi and algae from the Greenland Ice Sheet. Microb. Ecol. 86, 282–296. 10.1007/s00248-022-02033-5 35608637 PMC10293465

[B49] Remias D. Schwaiger S. Aigner S. Leya T. Stuppner H. Lütz C. (2012). Characterization of an UV- and VIS-absorbing, purpurogallin-derived secondary pigment new to algae and highly abundant in *Mesotaenium berggrenii* (Zygnematophyceae, Chlorophyta), an extremophyte living on glaciers. FEMS Microbiol. Ecol. 79, 638–648. 10.1111/j.1574-6941.2011.01245.x 22092588

[B50] Reuter J. A. Spacek D. V. Snyder M. P. (2015). High-throughput sequencing technologies. Mol. Cell 58, 586–597. 10.1016/j.molcel.2015.05.004 26000844 PMC4494749

[B51] Salmela L. Rivals E. (2014). LoRDEC: Accurate and efficient long read error correction. Bioinformatics 30, 3506–3514. 10.1093/bioinformatics/btu538 25165095 PMC4253826

[B52] Sánchez-Navarro R. Nuhamunada M. Mohite O. S. Wasmund K. Albertsen M. Gram L.. (2022). Long-read metagenome-assembled genomes improve identification of novel complete biosynthetic gene clusters in a complex microbial activated sludge ecosystem. mSystems 7, e0063222. 10.1128/msystems.00632-22 36445112 PMC9765116

[B53] Schaffer J. E. Reck M. R. Prasad N. K. Wencewicz T. A. (2017). β-Lactone formation during product release from a nonribosomal peptide synthetase. Nat. Chem. Biol. 13, 737–744. 10.1038/nchembio.2374 28504677

[B54] Scott T. A. Piel J. (2019). The hidden enzymology of bacterial natural product biosynthesis. Nat. Rev. Chem. 3, 404–425. 10.1038/s41570-019-0107-1 32232178 PMC7104373

[B55] Seemann T. (2014). Prokka: Rapid prokaryotic genome annotation. Bioinformatics 30, 2068–2069. 10.1093/bioinformatics/btu153 24642063

[B56] Shannon P. Markiel A. Ozier O. Baliga N. S. Wang J. T. Ramage D.. (2003). Cytoscape: a software environment for integrated models of biomolecular interaction networks. Genome Res. 13, 2498–2504. 10.1101/gr.1239303 14597658 PMC403769

[B57] Sim ao F. A. Waterhouse R. M. Ioannidis P. Kriventseva E. V. Zdobnov E. M. (2015). BUSCO: assessing genome assembly and annotation completeness with single-copy orthologs. Bioinformatics 31, 3210–3212. 10.1093/bioinformatics/btv351 26059717

[B58] Söderberg J. J. Grgic M. Hjerde E. Haugen P. (2019). Aliivibrio wodanis as a production host: development of genetic tools for expression of cold-active enzymes. Microb. Cell Fact. 18, 1–16. 10.1186/s12934-019-1247-1 31711487 PMC6844050

[B59] Soldatou S. Eldjárn G. H. Ramsay A. van der Hooft J. J. Hughes A. H. Rogers S.. (2021). Comparative metabologenomics analysis of polar actinomycetes. Mar. Drugs 19, 1–21. 10.3390/md19020103 33578887 PMC7916644

[B60] Stevens I. T. Irvine-Fynn T. D. L. Edwards A. Mitchell A. C. Cook J. M. Porter P. R.. (2022). Spatially consistent microbial biomass and future cellular carbon release from melting Northern Hemisphere glacier surfaces. Commun. Earth Environ. 3, 275. 10.1038/s43247-022-00609-0

[B61] Stintzi A. Johnson Z. Stonehouse M. Ochsner U. Meyer J. M. Vasil M. L.. (1999). The pvc gene cluster of *Pseudomonas aeruginosa*: Role in synthesis of the pyoverdine chromophore and regulation by PtxR and PvdS. J. Bacteriol. 181, 4118–4124. 10.1128/JB.181.13.4118-4124.1999 10383985 PMC93907

[B62] Terlouw B. R. Blin K. Navarro-Mu noz J. C. Avalon N. E. Chevrette M. G. Egbert S.. (2023). MIBiG 3.0: a community-driven effort to annotate experimentally validated biosynthetic gene clusters. Nucleic Acids Res. 51, D603–D610. 10.1093/nar/gkac1049 36399496 PMC9825592

[B63] Tian Y. Li Y. L. Zhao F. C. (2017). Secondary metabolites from polar organisms. Mar. Drugs 15, 28. 10.3390/md15030028 28241505 PMC5367009

[B64] Tracanna V. de Jong A. Medema M. H. Kuipers O. P. (2017). Mining prokaryotes for antimicrobial compounds: from diversity to function. FEMS Microbiol. Rev. 41, 417–429. 10.1093/femsre/fux014 28402441

[B65] Uritskiy G. V. DiRuggiero J. Taylor J. (2018). MetaWRAPa flexible pipeline for genome-resolved metagenomic data analysis. Microbiome 6, 158. 10.1186/s40168-018-0541-1 30219103 PMC6138922

[B66] Vanittanakom N. Loeffler W. Koch U. Jung G. (1986). Fengycin-a novel antifungal lipopeptide antibiotic produced by bacillus subtilis F-29-3. J. Antibiot. 39, 888–901. 10.7164/antibiotics.39.888 3093430

[B67] Vila E. Hornero-Méndez D. Azziz G. Lareo C. Saravia V. (2019). Carotenoids from heterotrophic bacteria isolated from Fildes Peninsula, King George Island, Antarctica. Biotechnol. Rep. 21, e00306. 10.1016/j.btre.2019.e00306 30705834 PMC6348148

[B68] Wanarska M. Krajewska-Przybyszewska E. Wicka-Grochocka M. Cieśliński H. Pawlak-Szukalska A. Bialkowska A. M.. (2022). A new expression system based on psychrotolerant debaryomyces macquariensis yeast and its application to the production of cold-active β-d-galactosidase from paracoccus sp. 32d. Int. J. Mol. Sci. 23, 11691. 10.3390/ijms231911691 36232994 PMC9569826

[B69] Wehrmann L. M. Riedinger N. Brunner B. Kamyshny A. Hubert C. R. Herbert L. C.. (2017). Iron-controlled oxidative sulfur cycling recorded in the distribution and isotopic composition of sulfur species in glacially influenced fjord sediments of west Svalbard. Chem. Geol. 466, 678–695. 10.1016/j.chemgeo.2017.06.013

[B70] Wick R. R. Schultz M. B. Zobel J. Holt K. E. (2015). Bandage: interactive visualization of de novo genome assemblies. Bioinformatics 31, 3350–3352. 10.1093/bioinformatics/btv383 26099265 PMC4595904

[B71] Wu Y.-W. Simmons B. A. Singer S. W. (2016). MaxBin 2.0: an automated binning algorithm to recover genomes from multiple metagenomic datasets. Bioinformatics 32, 605–607. 10.1093/bioinformatics/btv638 26515820

