# Supplementary figures and images for: The undiscovered biosynthetic potential of the Greenland Ice Sheet microbiome
Source: Front Microbiol. 2023 Dec 12;14:1285791. doi: 10.3389/fmicb.2023.1285791 (PMC10749974; doi:10.3389/fmicb.2023.1285791)

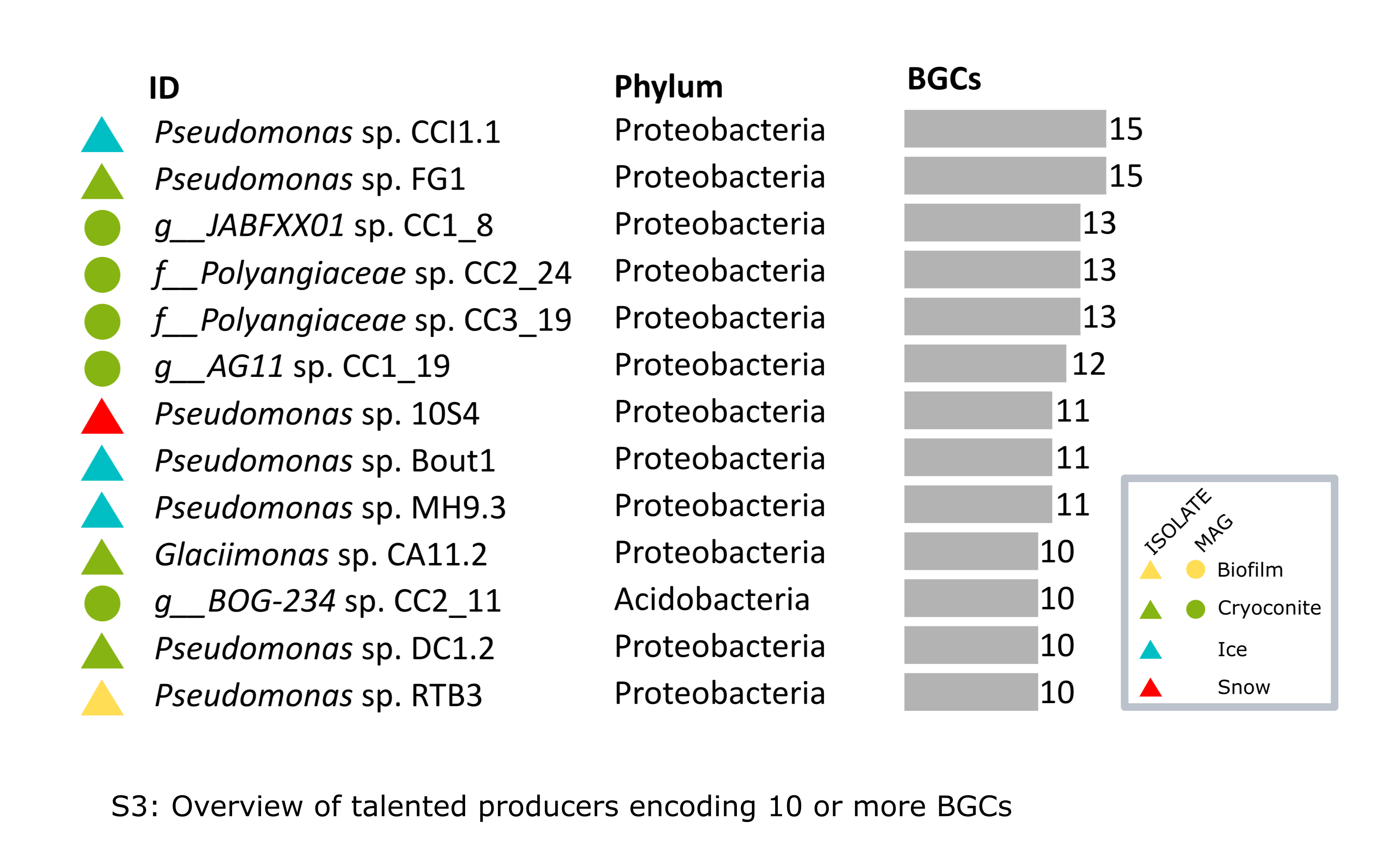

Supplement: Supplementary file 3 [file Image_1.PNG]

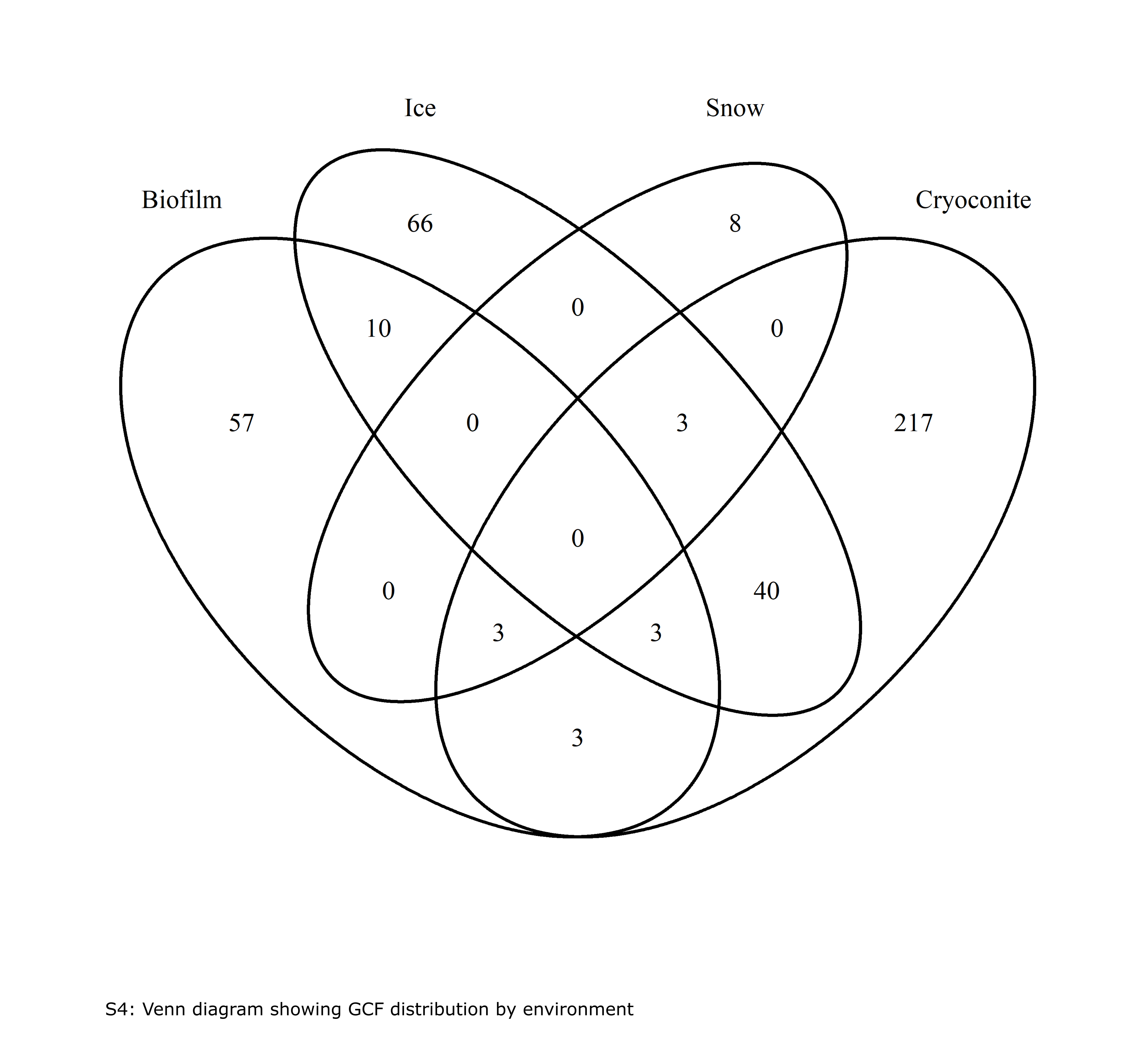

Supplement: Supplementary file 4 [file Image_2.PNG]

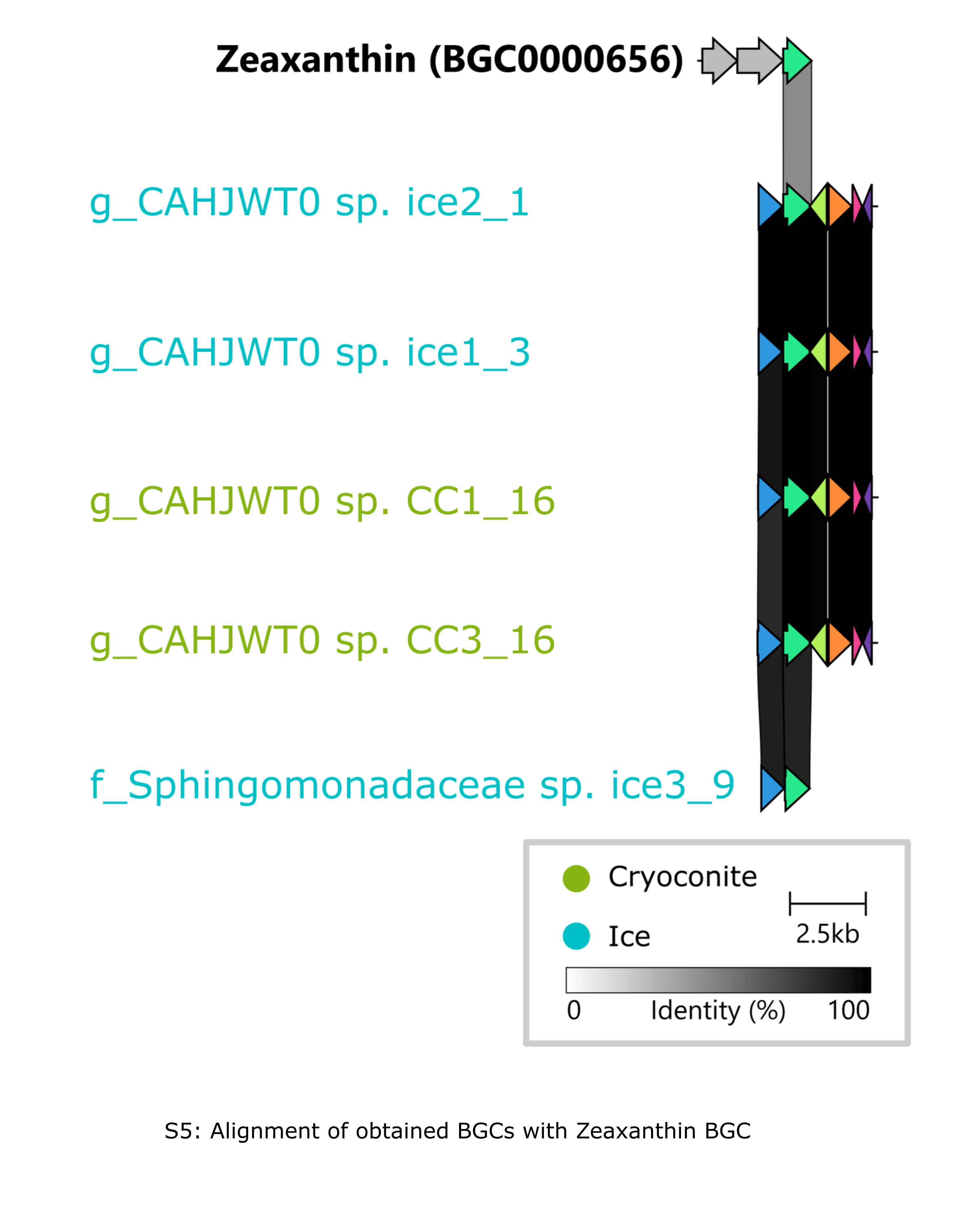

Supplement: Supplementary file 5 [file Image_3.PNG]
